# Repeated intraocular crystallization of ganciclovir in one eye after bilateral intravitreal injections: a case report

**DOI:** 10.1186/s12886-018-0703-8

**Published:** 2018-02-09

**Authors:** Lawrence P. L. Iu, Michelle C. Y. Fan, Wai-Ching Lam, Ian Y. H. Wong

**Affiliations:** Department of Ophthalmology, The University of Hong Kong, Grantham Hospital, 125 Wong Chuk Hang Road, Aberdeen, Hong Kong, Hong Kong

**Keywords:** Ganciclovir, Crystallization, Cytomegalovirus retinitis, CMV, Intraocular, Intravitreal, Precipitation

## Abstract

**Background:**

Cytomegalovirus (CMV) retinitis is an opportunistic infection that primarily affects immunocompromised individuals. Intravitreal ganciclovir injection monotherapy or in combination with systemic anti-CMV therapy are effective treatments for CMV retinitis. Crystallization of ganciclovir after intravitreal injection is extremely rare. Only two cases had been reported in literature. Crystallization in only one eye after bilateral injections had not been reported before. We hereby report a case of intraocular ganciclovir crystallization in one eye after bilateral intravitreal injections, and repeated crystallization in the same eye after repeated injections.

**Case presentation:**

A 79-year-old patient had bilateral cytomegalovirus retinitis and received bilateral intravitreal ganciclovir injections of 2.5 mg in 0.05 ml sterile water. Fundus examination after injection showed formation of needle-shaped, golden-yellow crystals in the vitreous of right eye but not in left eye. The crystals dissolved spontaneously. Repeated bilateral intravitreal ganciclovir injections 4 days later resulted in repeated crystallization of ganciclovir in right eye but not in left eye. The crystals dissolved spontaneously and completely after 5 minutes. Visual acuity remained unchanged and intraocular pressure was normal.

**Conclusions:**

Intraocular ganciclovir crystallization could occur after intravitreal injections. It is important to perform fundus examination after injection. The crystals may dissolve rapidly and vitrectomy may not be necessary. Our case suggested intraocular ganciclovir crystallization is an idiosyncratic phenomenon, subjects to distinctive intraocular environment which could be different between two eyes of the same patient. The susceptible intraocular environment could be persistent leading to repeated crystallization.

## Background

Cytomegalovirus (CMV) retinitis is an opportunistic infection that primarily affects immunocompromised individuals. Intravitreal ganciclovir injection monotherapy or in combination with systemic anti-CMV therapy are effective treatments for CMV retinitis [1–3]. Crystallization of ganciclovir after intravitreal injection is extremely rare. Only two cases had been reported in literature [4, 5]. Crystallization in only one eye after bilateral injections had not been reported before. We hereby report a case of intraocular ganciclovir crystallization in one eye after bilateral intravitreal injections, and repeated crystallization in the same eye after repeated injections.

## Case presentation

**Fig. 1:**
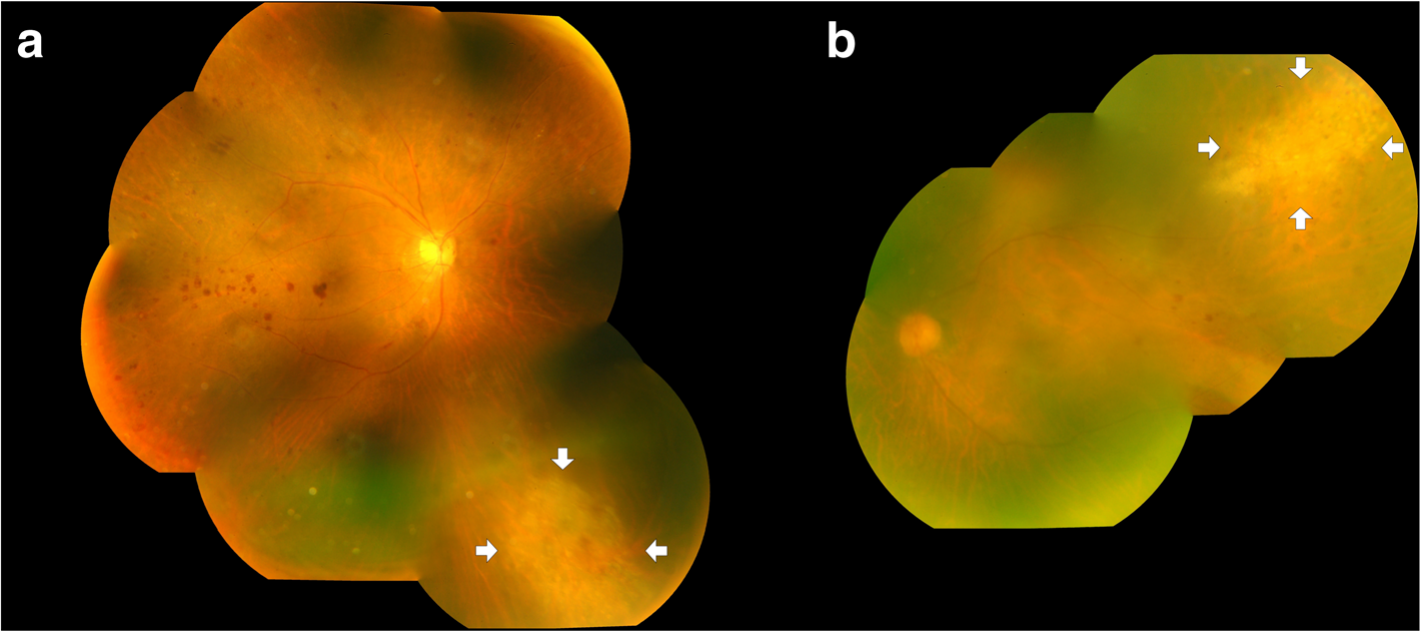
Fundus photography showing bilateral cytomegalovirus retinitis with retinal infiltrates in (**a**) right inferonasal and (**b**) left superotemporal peripheral retinae

A 79-year-old male was referred to our institution for bilateral blurring of vision. He had multiple medical problems including diabetes mellitus, bronchiectasis, ischemic heart disease, bullous pemphigoid, carcinoma of prostate with bone metastases and end-stage renal failure. He was on long term oral steroid for the disease of bullous pemphigoid. His systemic medication included allopurinol 100 mg daily, calcium carbonate 1000 mg daily, aspirin 100 mg with glycine 45 mg daily, ferrous sulphate 300 mg daily, flutamide 250 mg three times daily, frusemide 80 mg twice daily, insulin 32 units in the morning and 10 units in the afternoon, pantoprazole 20 mg daily, potassium chloride 600 mg daily, senna 15 mg daily, simvastatin 10 mg daily and prednisolone 10 mg daily. On presentation, his visual acuity (VA) was 20/60 OD and 20/100 OS. Intraocular pressure (IOP) was 11 mmHg OD and 10 mmHg OS. Slit-lamp examination showed mild anterior chamber cells and moderate cataract bilaterally. Fundus examination showed retinal infiltrates in right inferonasal and left superotemporal peripheral retinae (Fig. 1). There was bilateral mild vitreous haze and vessels detail was still visible (binocular indirect ophthalmoscopy score of 1). Diagnostic aqueous tap was performed for both eyes, which showed presence of CMV DNA in both eyes by polymerase chain reaction. Varicella-zoster virus and herpes simplex virus DNA were absent. A diagnosis of bilateral CMV retinitis was made. His white blood cell count was elevated at 12.8 × 10^9^/L (normal range 3.8–9.9 × 10^9^/L). He was anemic with blood hemoglobin count at 3.5 g/dL (normal range 4.4–5.7 g/dL). Liver and renal functions were deranged.

In view of the problem of CMV retinitis, oral valganciclovir was initiated at 450 mg on alternate days. Intravitreal ganciclovir treatment of 2.5 mg in 0.05 ml sterile water was given to both eyes. The ganciclovir solution was prepared by our institution’s pharmacy in two separate syringes for separate eye injections. Fundus examination after injections showed formation of needle-shaped, golden-yellow crystals in vitreous of right eye but not in left eye. He did not report any ocular pain or visual change. The crystals dissolved spontaneously.

**Fig. 2:**
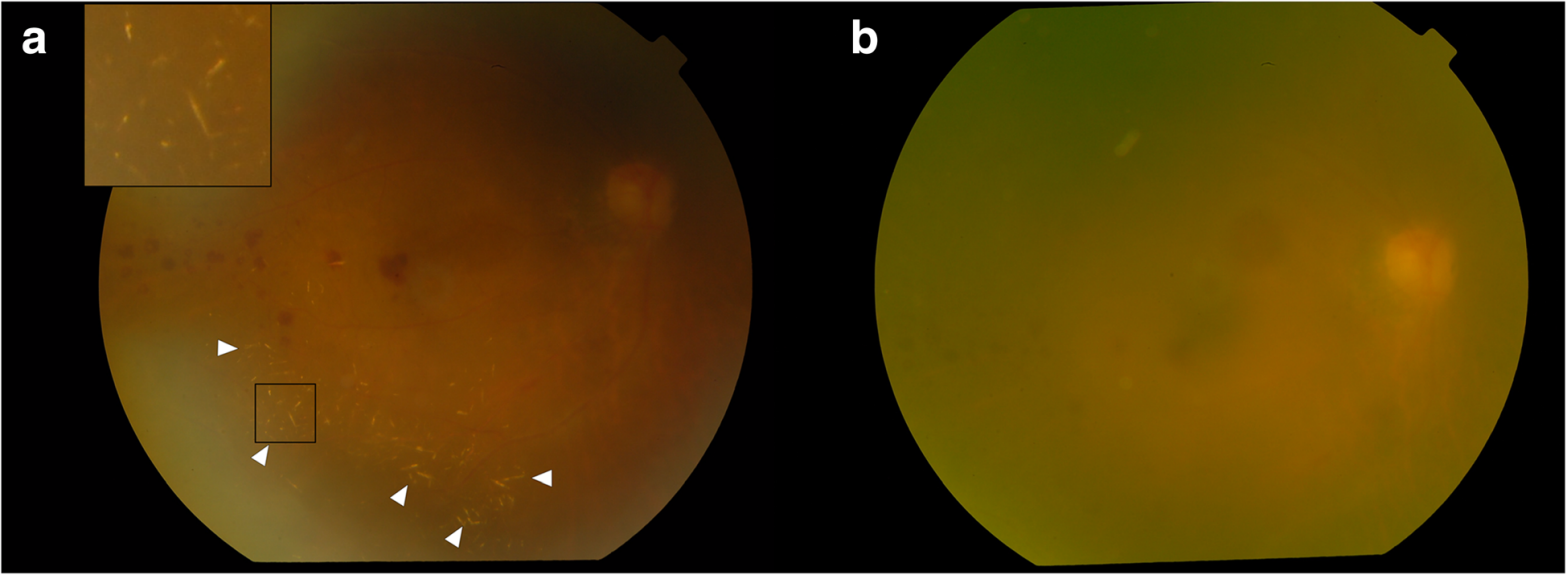
Fundus photography of right eye. **a** After intravitreal ganciclovir injection, needle-shaped, golden-yellow crystals were formed in the vitreous (arrowheads). Inset showing magnified view of the crystals. **b** The crystals dissolved spontaneously after 5 minutes

The patient was reviewed 4 days later, his VA was 20/600 OD and 20/100 OS. There were no signs of retinal vascular occlusion or optic neuropathy. Intravitreal ganciclovir injections of 2.5 mg in 0.05 ml sterile water were repeated. The ganciclovir solution was prepared by our institution’s pharmacy and had been checked to ensure it did not contain any crystals before injections. Crystallization of ganciclovir was noted again in right eye (Fig. 2a) but not in left eye after injections. He did not report any visual change. VA remained unchanged and IOP was normal. The crystals dissolved spontaneously after 5 minutes (Fig. 2b). There were no signs of retinal vascular occlusion. No further intravitreal ganciclovir treatment was given because his general condition deteriorated with sepsis, pneumonia and peritonitis. He passed away 1 month later.

## Discussion

Ganciclovir is a synthetic analogue of guanine which, after phosphorylation by CMV-encoded enzymes in infected cells, inhibits viral DNA synthesis and viral replication [6]. Ganciclovir is commercially available as lyophilized powder of ganciclovir sodium in strength of 500 mg per vial [6]. It is normally reconstituted with 10 ml of sterile water for injection to yield a solution with concentration of 50 mg/ml and an alkaline pH of 11 [6]. Ganciclovir sodium has an aqueous solubility of greater than 50 mg/ml at room temperature of 25 °C, and a much lower aqueous solubility of 6 mg/ml at physiological pH under body temperature of 37 °C [6]. The reconstituted solution is stable at room temperature for 12 h [6]. Crystallization of ganciclovir could occur if the reconstituted solution is exposed to temperature other than room temperature. In a previous report, crystals were observed in syringes after the reconstituted solution had been stored for 24 h in refrigerator at 4 °C, and the crystals dissolved within 4 h at room temperature [7]. It is also known that precipitation would occur if bacteriostatic water containing the preservatives parabens is used for reconstitution instead of sterile water [6].

In our case, the ganciclovir solution for intravitreal injection was prepared by institution pharmacy to avoid preparation errors. Sterile water was used for reconstitution and it did not contain any preservatives parabens. The solution was prepared within 4 h prior to intravitreal injection without being refrigerated or being exposed to change of temperature and therefore the solution should remain stable. This case suggests there are other mechanisms which give rise to intraocular crystallization of ganciclovir.

Intraocular crystallization of ganciclovir after intravitreal injection had been reported in only two cases in literature. In first report [4], crystallization occurred because the ganciclovir was prepared inadvertently at a high dose of 40 mg in 0.1 ml solution and resulted in retinal ischemia and necrosis. In a normal vitreous of around 4 ml volume and physiological pH, such a highly-concentrated ganciclovir solution would precipitate out and crystallize. Vitrectomy was performed to remove the crystals in that case [4]. In second report [5], crystallization occurred after intravitreal injection of ganciclovir at a dose of 4 mg in 0.04 ml solution which resulted in central retinal artery occlusion and optic neuropathy. Vitrectomy was performed to remove the crystals [5]. The authors postulated that sudden change of osmolarity or pH in the globe after injection played a role in crystallization [5]. In both cases, patients had immediate drop of vision and acute rise of IOP after injections [4, 5].

The needle-shaped, golden-yellow ganciclovir crystals in our case resembled those reported in literature [4, 5]. Our patient, however, had spontaneous rapid dissolution of crystals and did not have any signs of vascular occlusion or acute rise of IOP. The exact mechanism of crystallization is unknown. Our case concurred with previous authors’ postulation that sudden change of osmolarity or pH in the globe after injection might contribute [5]. Interestingly, our case also suggested intraocular ganciclovir crystallization is an idiosyncratic phenomenon, subjects to distinctive intraocular environment which could be different between two eyes of the same patient. The susceptible intraocular environment could persist and result in repeated crystallization with repeated injections. We also speculated that the volume of vitreous cavity and amount of vitreous syneresis were attributable factors because they could influence the distribution and dispersion of ganciclovir in vitreous. Uneven distribution in vitreous cavity could result in more concentrated ganciclovir in some parts of vitreous that exceeds aqueous solubility.

Foscarnet is another anti-CMV drug which is often used intravenously and intravitreally for CMV retinitis. In a previous case report [8], foscarnet crystals appeared in the vitreous after 11 intravitreal injections of foscarnet (2.4 mg in 0.1 ml) were given within 2 months. Similar to our case of ganciclovir crystallization, there was no evidence of retinal or optic nerve damage [8]. The authors postulated that foscarnet crystallization was caused by sudden change of vitreous pH after injections [8].

The major limitation of this case report is that it consists of a single case only, therefore causal-effect relationship was difficult to interpret and the clinical features might not be representative. In addition, due to the poor general condition of patient, the number of intravitreal treatments was limited. Investigations such as fluorescein angiography and optical coherence tomography were also not performed. Despite these limitations, this case report added a new observation of intraocular ganciclovir crystallization which occurred repeatedly in the same eye after bilateral injections.

## Conclusions

In conclusion, intraocular ganciclovir crystallization could occur after intravitreal injections and could be related to distinctive intraocular environment. It is important to perform fundus examination after injection. The crystals may dissolve rapidly and vitrectomy may not be necessary. Further studies are warranted to investigate the mechanism for intraocular ganciclovir crystallization.

## Abbreviations

CMV: Cytomegalovirus
IOP: Intraocular pressure
VA: Visual acuity
